# EMG and kinematic analysis of sensorimotor control for patients after stroke using cyclic voluntary movement with visual feedback

**DOI:** 10.1186/1743-0003-10-18

**Published:** 2013-02-08

**Authors:** Rong Song, Kai Yu Tong

**Affiliations:** 1 School of Engineering, Sun Yat-sen University, Guangzhou, Guang Dong, P. R. China; 2 Interdisciplinary Division of Biomedical Engineering, the Hong Kong Polytechnic University, Rm ST417, Core S, 4/F, Kowloon, Hong Kong

## Abstract

**Background:**

Clinical scales are often used to evaluate upper-limb deficits. The objective of this study is to investigate the parameters during voluntary arm tracking at different velocities for evaluating motor control performance after stroke.

**Methods:**

Eight hemiplegic chronic stroke subjects were recruited to perform voluntary movements of elbow flexion and extension by following sinusoidal trajectories from 30 deg to 90 deg at six velocities in the horizontal plane by completing 3, 6, 8, 12, 15, 18 flexion and extension cycles in 36 seconds in a single trial, and the peak velocities ranged from 15.7 to 94.2 deg/s. The actual elbow angle and the target position were displayed as real-time visual feedback. The angular displacement of the arm and electromyographic (EMG) signals of biceps and triceps were captured to evaluate the sensorimotor control of the affected and unaffected side.

**Results:**

The results showed significant differences in the root mean square error (RMSE), response delay (RD) and cocontraction index (CI) when the affected and unaffected sides were compared during the arm tracking experiment (P<0.05). RMSE decreased with the increase in the tracking velocities for the affected and unaffected sides. And CI and RD increased with the increase in the tracking velocities for both sides. There was significant correlation between average RMSE of the six velocities and Fugl-Meyer shoulder-elbow score for the eight poststroke subjects.

**Conclusions:**

The method and parameters have potential for clinical use in quantitatively evaluating the sensorimotor deficiencies for patients after stroke about the accuracy of motion, response delay and cocontraction between muscle pairs.

## Background

Stroke is the leading cause of disabilities in China and many other countries and rehabilitation is important for motor function recovery to facilitate patients after stroke back to normal activities of daily life [1]. Recent studies suggested patients after stroke should conduct different kinds of therapeutic interventions when they are in different stages of motor status [2]. In order to apply suitable treatment strategies for persons after stroke, it is important to understand the deficiencies induced by stroke and the progress achieved through rehabilitation therapy.

Clinical scales such as Ashworth scale and Fugl-Meyer assessment are often used to evaluate upper-limb deficits [3,4]. However, the clinical scales are semi-quantitative methods, which may not be sensitive enough to detect gradual muscle progress and motor coordination changes during the rehabilitation process [5]. More quantitative ways have been sought to evaluate the affected joints in patients after stroke, such as passive mechanical properties during the constant velocity stretch test [6-8], the sinusoidal excitation test [9,10], and the pendulum test [11]. Kinematic analysis of subjects after stroke is also an important tool to evaluate the motor disorder during voluntary movement. A number of invariant features of single-joint movements have been observed from the trajectories that the plan of movements appears to be independent of the subjects, in which a limb has symmetric, bell-shaped velocity profiles in single-joint movements. Hogan (1984) proposed a principle underlying the selection of a movement trajectory by the central nervous system (CNS) [12]. The movement with maximum smoothness is most likely to be selected among all possible trajectories. Wiegner et al. investigated a seventh-order polynomial minimum-snap model, which was an extension of the five order minimum-jerk model and was consistent with the physiological range of the rate of change of the torque [13]. Mescheriakov et al. also proposed that the acceleration-time profile of the movement can be described by a linear combination of two Gaussian functions (positive for acceleration and negative for deceleration) [14]. Feng et al. investigated the spastic elbow movement in three-dimensional (3D) space [15]. In 1954, the Information theory was employed to explain the human motor system by Fitts et al., who mathematically integrated speed, accuracy, amplitude of the movements and target size into a one-dimensional parameter to evaluate upper extremity tasks [16]. McCrea et al. studied the stroke-induced changes to motor control of the affected arms of subjects after stroke. The study quantified the capacity of CNS transmitting motor commands by a linear relationship between movement time and task difficulty (Fitts’ law) during a reaching task. They compared the affected arm of 20 persons after stroke with the non-dominant arm of ten healthy persons. The results found that there were significantly increases of Fitt’s slope and intercept in the more affected arms of the group with stroke. Indirect, segmented, and positively skewed movement was found in the group with stroke, which could result from greater neuromotor noise [17].

EMG and kinetic measures have been used as the primary tools in the study of movement, which provide an electrophysiological view of movement. The methods are also used to analyze the motion disorder after stroke. Canning et al. investigated the abnormalities of muscle activation with low dexterity after stroke. They found excessive biceps muscle activation and decreased coupling of muscle activation to target motion. Weakness, slowness of muscle activation, excessive co-contraction, and spasticity can cause the abnormalities after stroke [18]. Chae et al. recorded EMG activity of the paretic and non-paretic wrist flexors and extensors from 26 chronic stroke survivors during isometric wrist flexion and extension in order to find the relationship between post-stroke upper limb muscle weakness, co-contraction, and clinical measures of upper limb motor impairment and physical disability [19]. In their research, they found that the strength of muscle contraction was significantly greater in the non-paretic limb; the degree of co-contraction was significantly greater in the paretic limb; muscle weakness and degree of co-contraction correlated significantly with motor impairment and physical disability in upper limb hemiplegia. They also found that delay in initiation and termination of muscle contraction was significantly prolonged in the paretic arm and the delay did not have significant correlation with motor impairment and physical disability [20]. Dickstein et al. found that EMG activity of rectus abdominis was significantly delayed in comparison to that of external oblique relative to the unaffected side in the patients and relative to the control subjects during voluntary trunk flexion [21].

However, these studies focused on motor execution and did not include the sensory feedback, which was also an important source for the central nervous system to correct and coordinate the movement. The main objective of this paper was to quantitatively evaluate the elbow sensorimotor control ability of subjects after stroke during the voluntary tracking task, which coupled the sensory and motor functions of the neuromusculoskeletal system in order to comprehensively analyze the disorder caused by stroke. A sinusoidal tracking trajectory was designed, because the velocity profile was similar to the bell-shaped velocity profile in single-joint movement of human. In this study, arm tracking test was design to evaluate stroke-induced deficiencies in sensorimotor control of affected elbow, and low inertia and ignorable friction torque of the system could minimize external interface to the voluntary movement.

## Methods

Eight subjects (six males and two females) after stroke were recruited in this study. The mean age of the subjects was 45±11 years and the range was from 21 to 57 years. Table 1 summarized the basic clinical information, modified Ashworth scale, Fugl-Meyer wrist-hand score, and Fugl-Meyer shoulder-elbow score of all the subjects. The subject selection criteria included: (1) hemiparesis resulting from a single unilateral lesion of the brain with onset at least six months before data collection; (2) active elbow range of motion (ROM) was 30 deg-90 deg on the affected side; and (3) subjects should not have any medical history of visuospatial, cognitive or attention deficits, and they could understand instructions and perform a screening test at the tracking velocity of 47.1 deg/s by following the target. This study was reviewed and approved by the local university human ethical committee. Before the test, the experimental protocol was introduced to all the subjects, and they gave their informed consent following the ethical procedures.

**Table 1:** Clinical data from the subjects after stroke

**Subject**	**Age/ (Sex)**	**Lesion side**	**Years after stroke**	**Fugl-Meyer score (S/E)**	**Fugl-Meyer score (W/H)**	**Modified Ashworth scale**
A	37 (M)	R	11 yrs	15	5	2
B	45 (F)	L	2 yrs	15	2	1
C	51 (F)	L	1 yr	12	6	1+
D	52 (M)	R	4 yrs	20	3	1+
E	49 (M)	L	1yr	12	3	1
F	57 (M)	R	13 yrs	12	7	3
G	42 (M)	L	4 yrs	10	9	1+
H	60 (M)	R	5yrs	14	1	1+

**Figure 1:**
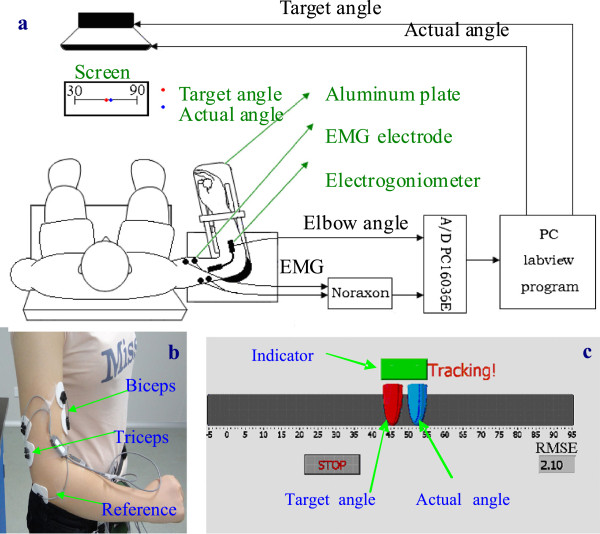
(a) Block diagram of experimental setup; (b) placement of the electrodes; (c) Labview interface for tracking.

In the experiment, the subjects were instructed to sit beside the table. A strap was used to fix the upper arm to a supporter on the table. The height of the table was adjusted to rest the arm in the horizontal plane with the same height as that of the shoulder, and the shoulder was in 90 deg abduction and 45 deg horizontal flexion. The forearm was attached to a manipulandum with the axis of rotation in line with the elbow joint (Figure 1). The manipulandum was used to support the forearm. The rotation axis was connected with a ball bearing, and the friction torque along the rotation axis is negligible (less than 0.1Nm) with respect to the torque generated by the subjects. The manipulandum was made of aluminum and weighed about 400g. The design was to minimize the inertial effect from the manipulandum during the voluntary arm movement. A computer screen was placed in front of the subjects, which displayed both the target and the actual elbow angle. The subjects were instructed to initially set the elbow at 30 deg flexion, since many subjects after stroke often had difficulty moving to the fully extended position. After a random delay generated by the Labview software which ranged from 2 to 5 sec, the indicator light in the middle of the screen turned green, and the target pointer began to move along the horizontal line in a sinusoidal trajectory between 30 deg and 90 deg, and each trial was 36 seconds. The subjects were instructed to try their best to follow the moving target pointer by controlling their elbow angle. The actual elbow angle was also displayed in another pointer as the real-time feedback. Before the test, three warm-up trials were arranged for the subjects to get familiar with the experiment. Then each subject was administered 18 trials structured in three blocks. Each block consisted of six trials with different velocities, which were arranged in a random sequence. In each trial, subjects were ask to complete different number of cycles (3, 6, 8, 12, 15 and 18 cycles) of sinusoidal trajectory of flexion and extension movements in 36 seconds resulting in six different peak velocities (15.7, 31.4, 47.1, 62.8, 78.5, and 94.2 deg/s, respectively). The main goal is to track the target as close as possible to minimize the error. The subjects had a 30-second and 5-minute rest time between each trial and block respectively. For all the subjects, the task was performed on both the affected and unaffected arms. The angular displacement of the elbow joint was captured by a flexible electrogoniometer (Penny & Giles, UK), which was attached to the manipulandum. A tele-EMG system (MyoSystem1400, Noraxon, USA) with a bandwidth of 10–500 Hz per channel was used to capture and amplify the surface EMG signals from two selected muscles: biceps brachii and medial triceps brachii, which were the muscle groups that mainly contributed to the movements of elbow flexion and elbow extension. The surface EMG signals were captured with Ag/AgCl surface electrodes (Noraxon, USA). All Ag/AgCl electrodes were placed in bipolar configuration with a 2 cm space between the centers of the electrodes. The angle signal and EMG signals from biceps brachii and medial triceps brachii were recorded simultaneously at a sampling frequency of 1000 Hz and were stored in a PC via a 16-channel A-D converter for off-line analysis (PCI 6036E, National instrument, Texas, USA).

### Evaluation procedures

#### 1) Clinical scales

These scales included the Fugl-Meyer (range 0–66 for upper limb including shoulder-elbow score (0–42) and wrist-hand score (0–24)) [22,23] for the evaluation of motor function and the modified Ashworth scale (range 0–4) [24] for the muscle tone at the elbow joint.

#### 2) Root mean square error and response delay

RMSE evaluated the voluntary tracking performance of all subjects.

(1) $$\text{RMSE}={\left(\frac{\sum {\left(\theta_{0}\left(i\right)-\theta \left(i\right)\right)}^{2}}{N}\right)}^{1/2}$$

where *θ*_0_(*i*) was the target elbow angle at ith sampling instant and *θ*(*i*) was the actual elbow angle at ith sampling. N was the total number of samples.

The response delay (RD) was used to describe the time interval between the trajectory of the actual elbow and the trajectory of the target, which was quantified by the temporal shift (t) that maximized the following normalized cross-correlation function: [25]

(2) $$R_{\mathrm{xy}}\left(\tau \right)=\frac{\int_{-T}^{T}x\left(t\right)y\left(t+\tau \right)\mathrm{d\tau}}{R_{\mathrm{xx}}R_{\mathrm{yy}}}$$

where *R*_*xy*_ was the value of the cross-correlation between the target trajectory and the actual trajectory at any time shift *τ*. T was the length of the records, which equaled to the length of one cycle for each velocity in this experiment; x and y were the target and actual elbow angle in time domain; *dτ* was the interval between the adjacent time shifts and its resolution was 0.001s; *R*_*xx*_ and *R*_*yy*_ were the maximum values of the auto-correlations of the target and actual angle trajectories respectively, which were defined at *τ* = 0. The cross-correlation technique was adopted to calculate the RD, which avoided the subjective criteria for defining the onset of actual trajectory.

#### 3) Co-contraction index

The co-activations between triceps and biceps during tracking movement were studied using the co-contraction index (CI) as introduced in Frost’s study [26], shown in the following equation:

(3) $$\mathrm{CI}=\frac{1}{T}{\int {}_{T}\text{EMG}}_{\mathrm{bt}}\left(t\right)\mathrm{dt}$$

where EMGbt(t) is the overlapping activity of normalized envelopes for biceps and triceps, T is the length of the signal trial. The range of a CI was from 0 (nonoverlapping at all) to 1 (totally overlapping).

### Statistical analysis

All data were tested for normality with the Kolmogorov-Smirnov test. A two-way ANOVA with repeated measures was applied to statistically analyze the above three parameters (RMSE, RD and CI), which comprised of two main factors: side (affected or unaffected side) and tracking peak velocities (15.7, 31.4, 47.1, 62.8, 78.5, and 94.2 deg/s). The statistical model was used to analyze the main effects of side and velocity on the ROM, RD and CI. Testing of the difference between the affected and unaffected sides under the same velocity in terms of RMSE, RD and CI was performed with the paired t-test (two-tail test). The relationship between the parameters (RMSE, RD and CI) and clinical scales (Fugl-Meyer shoulder-elbow score, Fugl-Meyer wrist-hand score, Fugl-Meyer score for upper limb, and the modified Ashworth scale) were also investigated by using a cross-correlation coefficient. The significant level of all cases was set at 0.05. All statistical work was performed with SPSS 12. (SPSS Inc., Chicago, Illinois, USA).

## Results

**Figure 2:**
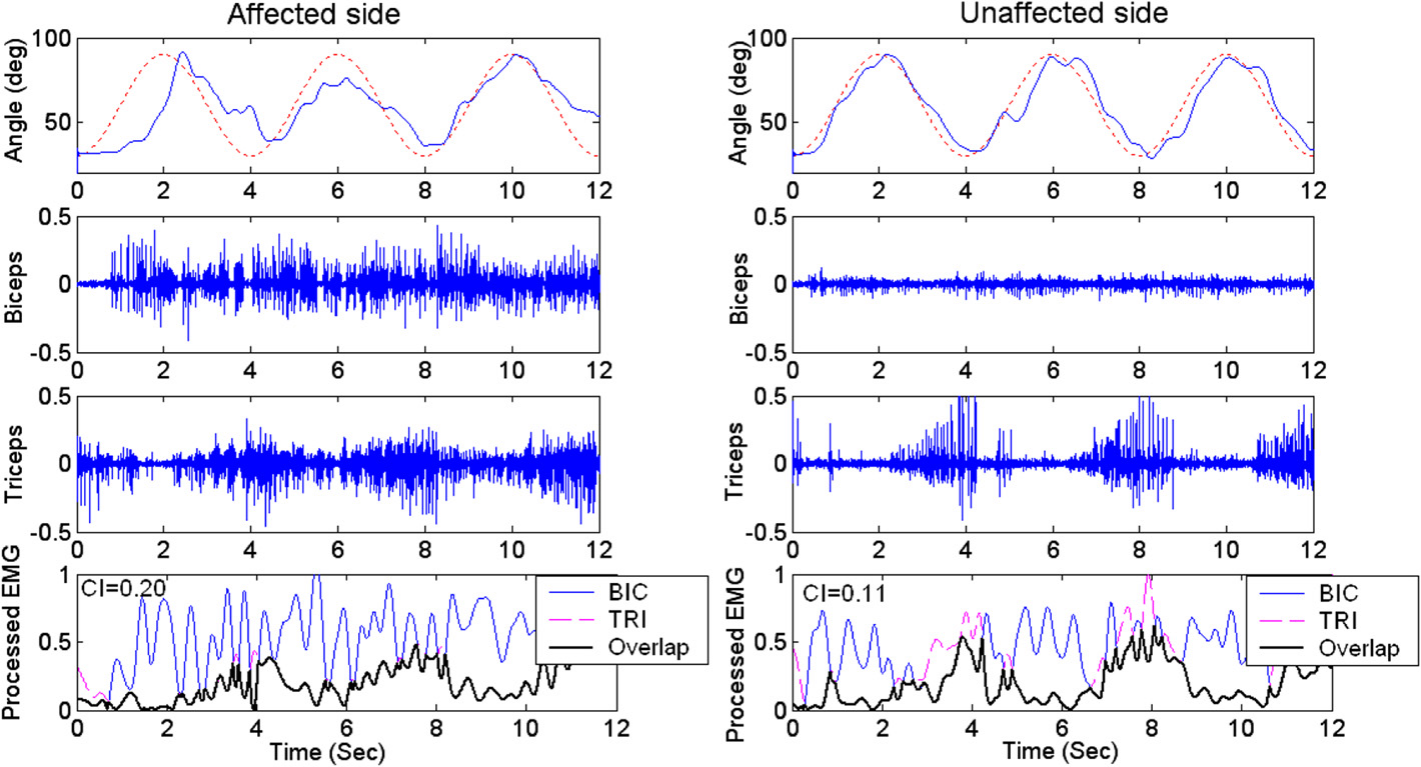
**The elbow trajectories, the EMG signals and CI. **The elbow trajectories (solid line), the EMG signals of biceps and triceps and CI of a subject during the voluntary elbow tracking at a velocity of 47.1 deg/s. The dashed line was the target trajectory (left column: affected side; right column: unaffected side).

**Figure 3:**
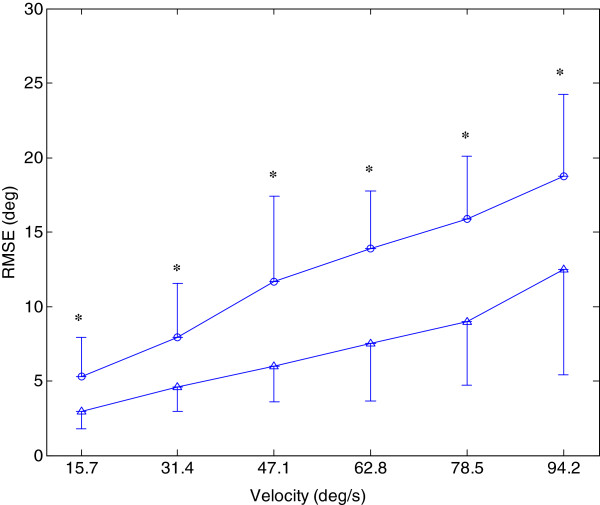
**Comparison between the average RMSE of the affected side and unaffected side. **Comparison between the average RMSE of the affected side (○) and unaffected side (Δ) at six velocities (15.7, 31.4, 47.1, 62.8, 78.5, and 94.2 deg/s) during the elbow tracking movement. Vertical bars indicate one standard deviation (* p<0.05).

The Distribution of outcome measures of RMSE, RD and CI were normal in both the affected (p=0.42) and unaffected side (p=0.10) by Kolmogorov-Smirnov test for normality. Figure 2 showed the trajectory and EMG signals of biceps and triceps when the voluntary movement was at a velocity of 47.1 deg/s Figure 3 plotted the comparison between the group mean RMSE of the unaffected side and that of the affected side at six velocities (15.7, 31.4, 47.1, 62.8, 78.5, and 94.2 deg/s). There was significant effect of the velocity and side on the range of motion for the unaffected and affected sides based on the two-way ANOVA with repeated measures (P<0.01). RMSE increased with the increase in the tracking velocity. Based on paired t-test, the average RMSE of the affected side was significantly larger than that of the unaffected side at all the velocities.

**Figure 4:**
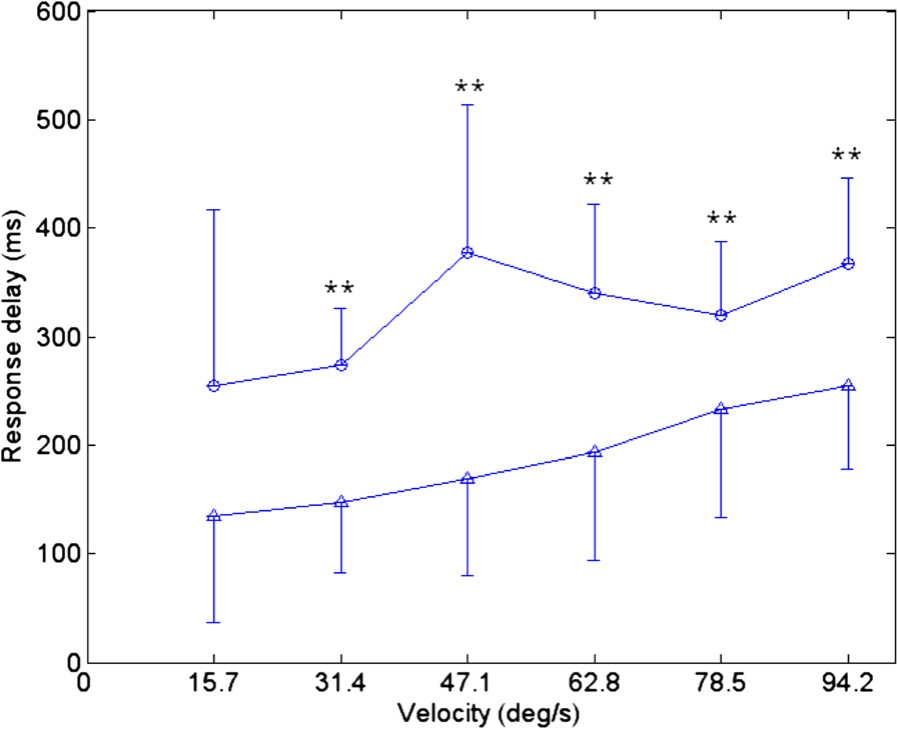
**Comparison between the average response delay of the affected side and unaffected side. **Comparison between the average response delay of the unaffected side (Δ) and affected side (○) at six velocities (15.7, 31.4, 47.1, 62.8, 78.5, and 94.2 deg/s) during the elbow tracking movement. Vertical bars indicate one standard deviation (* p<0.05, ** p<0.01).

The RD from all trials for both the affected side and the unaffected side were ranged from −195 to +495 ms and from 23 to 412 ms, respectively. The negative value implied that the phase of the actual elbow angle led the phase of the target angle. Figure 4 showed the comparison between the affected and unaffected sides at different tracking velocities. There was an increase in the RD for unaffected side with the increase in the tracking velocities. In the affected side at low velocities (15.7-47.1 deg/s), the RD had a larger variation among subjects, which could be reflected by the standard deviation. The actual elbow trajectory lagged behind the target trajectory in most of the trials, but there were three trials from two subjects in which the elbow trajectory led the target trajectory at the velocity of 15.7 deg/s. The two-way ANOVA with repeated measures showed that there was a significant difference between the affected side and the unaffected side (P<0.01). There was significant increase in RD of the affected side in comparison to the unaffected side at the velocities of 31.4, 47.1, 62.8, 78.5 and 94.2 deg/s (P<0.01) based on the paired t-test. For the velocities at 15.7, there was non-significant increase in the RD of the affected side in comparison to the unaffected side (P=0.074).

**Figure 5:**
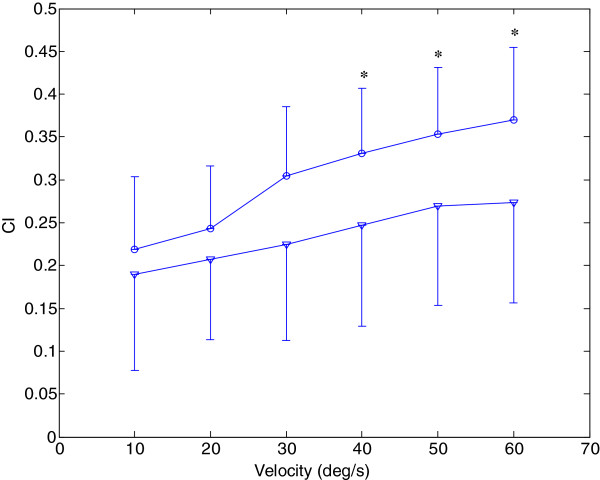
**Comparison between the average CI of the affected side and unaffected side. **Comparison between the average cocontraction index of the unaffected (∇) and affected sides (○) at six velocities (15.7, 31.4, 47.1, 62.8, 78.5, and 94.2 deg**/**s) during the elbow tracking movement. Vertical bars indicate standard deviation (* p<0.05, ** p<0.01).

Figure 5 showed the comparison of CI between the affected and unaffected side at different tracking velocities. There was an increase in CI for both sides with the increase in the tracking velocities. The two-way ANOVA with repeated measures showed that there was a significant difference between the affected side and unaffected side at different tracking velocities (P<0.01). Based on the paired t-test, there were significant increases in CI of the affected side in comparison to that of the unaffected side at three higher velocities (62.8, 78.5, and 94.2 deg/s), and there was non-significant increase in CI at three lower velocities (15.7, 31.4, and 47.1 deg/s).

**Figure 6:**
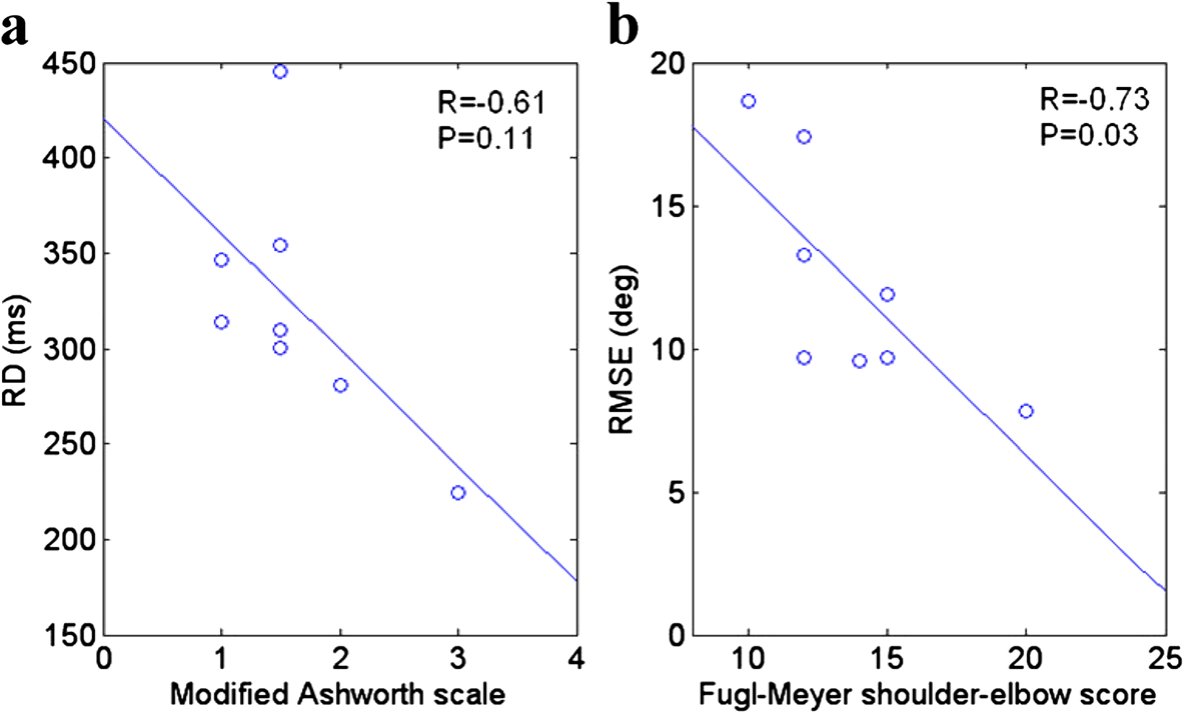
**(a) scatterplots of the modified Ashworth scale and the average RD of the six tracking velocities for eight poststroke subjects. **(**b**) Scatterplots of the Fugl-Meyer shoulder-elbow score and the average RMSE Solid line was the linear regression noted with the correlation coefficient, R and the statistical value, P.

Table 2 showed the correlation coefficients (adjusted R-Square) between the clinical scales (Fugl-Meyer upper limb score, Fugl-Meyer wrist-hand score, Fugl-Meyer shoulder-elbow score, the modified Ashworth scale) and the average parameters (RMSE, RD and CI) of the six velocities for eight poststroke subjects during elbow tracking movement. From the table, there was significant correlation between average RMSE and Fugl-Meyer shoulder-elbow score, and RD also showed a non-significant but strong correlation with the modified Ashworth scale (R= −0.61, P=0.11) (see Figure 6).

**Table 2:** The adjusted R-Square between the clinical scales ( Fugl-Meyer shoulder-elbow score, Fugl-Meyer wrist-hand score, Fugl-Meyer score for upper limb, modified Ashworth scale) and the average parameters of the six velocities (RMSE, RD and CI) for eight subjects after stroke during elbow tracking movement

**Parameter**	**Tracking velocities (deg/s)**			
	**Fugl-Meyer score (shoulder/elbow)**	**Fugl-Meyer score (wrist/hand)**	**Fugl-Meyer score for upper limb (shoulder/elbow + wrist-hand)**	**Modified Ashworth scale**
RMSE	0.47*	0.14	−0.04	−0.12
RD	0.04	0.32	−0.04	0.265
CI	−0.16	0.01	−0.07	−0.15

*P<.05.

## Discussion

The arm-tracking experiments were conducted to evaluate the sensorimotor control in a dynamic situation. In general, the kinematic profiles generated by the affected side had larger variability in different cycles (see Figure 2), which implies it is more difficult to keep consistent trajectories by CNS on the affected side. From Figure 2, the difficulty also could be reflected from abnormal co-contraction of agonist and antagonist. The subjects could accomplish the tracking task better at lower velocities when using their affected side, and they had difficulty in following the target at higher velocities which resulted in the increase in RMSE shown in Figure 3. RMSE could be used as a performance indicator to reflect overall sensory perception and motor action abilities. The RMSE values from the affected side were larger than those from the unaffected side, which implied that the damage in brain had affected both sensory perception and motor action abilities in the affected side. Patten et. al. [27] and Ju et. al. [28] evaluated the sensorimotor control in hemiparetic adults with elbow tracking task. In Patten’s study, subjects performed an elbow flexion and extension task against a low-resistance isotonic load at 3 speeds: 25, 45, and 65 deg/s from 10 deg of extension to 75 deg of flexion. The best performance occurred at a velocity of 45 deg/s for the affected side [27]. Ju et al. compared the tracking performance among three different loading conditions: no assistive or resistive loading. They found a non-significant decrease of RMSE in the affected sides when the external loading was applied [28]. The objective of this study was to minimize the external effect during cyclic voluntary movements, which was different from the above-mentioned studies in the following part: first, external torque was applied to the elbow in Patten and Ju’s work, which might have affected the voluntary tracking result; second, the moment of inertia of the systems in their studies and our previous study [29] might also have been considerable, which would inevitably affect the elbow voluntary movement; furthermore, the friction torque in the robotic systems might affect the voluntary elbow movement. The significant negative correlation between average RMSE with different tracking velocities and Fugl-Meyer shoulder-elbow score showed its relationship with clinical scores in quantitatively evaluation of motor function for patients after stroke. Since wrist and hand did not contribute to the elbow movement, there was no significant correlation between average RMSE with different tracking velocities and Fugl-Meyer wrist-hand score.

The RD in this study represented the overall delay throughout the full cycle, which was related with the time needed for CNS to receive sensory information, process the information and send motor command to muscle. The RD was significantly longer in the affected side than the unaffected side and this could be related to lesions which caused specific impairments in the afferent processing and efferent mechanisms of CNS. This finding was consistent with the report of other studies which concluded that there was a significantly longer initial and termination of the muscle force in the affected wrist [20] and hand [30] than the unaffected sides. In our study, during the low velocity tracking (15.7 deg/s), a large deviation in the delay was found among subjects (see Figure 4); some had lags and some had advances between the target and the actual trajectory. When the velocity increased, it required faster response for subject to follow the target in a cycle; therefore it was harder for the subjects to follow the trajectory which resulted in an increase in the RD. When the tracking velocity was from 31.4 deg/s to 94.2 deg/s, both RD from affected side and unaffected side increase. Although there was no significant correlation between the modified Ashworth scale and RD, the correlation was strong indicating that muscle tone may be one of important factors result in longer initial of movement.

There was no significant correlation between clinical scales and CI, the significant difference between the affected and unaffected side provide information on the muscle coordination and contraction between muscle pairs. There was an increase in CI with the increase in the tracking velocities. The significant increase in CI of the affected sides might be explained by two reasons: the increase of the EMG activation level of biceps and triceps, and the increased cocontraction phase of these two muscles. With the increase of velocities, both the activation level of agonist and antagonist increase, which resulted in the increase of CI in both sides. The change in CI in the affected side reflected the impairment of the ability to selectively activate flexor and extensors [31], and resulted in an increase of cocontraction phase between biceps and triceps. This often occurred in biceps activation during elbow extension, while triceps was less activated during elbow flexion. This was consistent with the EMG activation mode found in our study (shown in Figure 2). Accurate and smooth trajectory is planned by central nervous system for optimal control of arm movements in healthy subject, and minimal muscle activities is needed during task performance [32]. The proper cocontraction between agonist and antagonist muscle pair from the unaffected side could help to stabilize the joint and result in above-mentioned optimal control [33], while excessive cocontraction between agonist and antagonist from the affected side reflects the loss of optimal control of muscle activities, and results in a neither accurate nor an energy-saving control [34]. Dewald et al. reported abnormal muscle coactivating pattern at the elbow and shoulder in hemiparetic subjects [35]. Loss of supraspinal inhibitory function has been found in subjects after stroke [36,37]. Kisiel-Sajewicz also found the weakening of synergist muscle coupling during reaching movement in stroke patients [38]. Our previous research found that the elbow control function improved associated with the decrease of CI between biceps and triceps during robot-assisted rehabilitation [39], which implied the parameter might be a useful tool to evaluate how CNS coordinates flexor and extensor during voluntary movement.

There is a limitation of the current study is the effect of dominant and non-dominant side after stroke has not been considered, which is a factor need to be considered in the future study.

## Conclusions

This study investigated how stroke-induced sensorimotor deficiencies affected the motor control performance during a tracking task with various velocities. RMSE reflected the overall performance of sensory-motor control and was significantly related with Fugl-Meyer scale. RD also showed a non-significant but strong correlation with the modified Ashworth scale. These two parameters can quantitatively described the sensorimotor deficiencies with any measurement device that is capable of measuring elbow joint angle. There was no significant correlation between clinical scales and CI, the significant difference between the affected and unaffected side provide information on the muscle coordination and contraction between muscle pairs.

## Competing interests

The authors declare that they have no competing interests.

## Authors’ contributions

RS designed the study and carried out the experiment. Both KT and RS analyzed the data, interpreted the results, drafted and revised the manuscript. All authors approved the final version of the manuscript.

## Authors’ information

**Rong Song** received the B.S. degree in electrical engineering from Tsinghua University, Beijing, China, in 1999, the M.S. degree in electronic engineering from Shantou University, Shantou, China, in 2002, and the Ph.D. degree in biomedical engineering from the Hong Kong Polytechnic University, Kowloon, Hong Kong, in 2006. He is currently associate professor in school of engineering, Sun Yat-sen University, P.R. China. His research interests include musculoskeletal modeling, biomedical signal processing, human motion analysis, and robot-assisted stroke rehabilitation.

**Kai-yu Tong** received the Ph.D. degree in bioengineering from the University of Strathclyde, Glasgow, U.K., in 1998. He spent four months as a Research Fellow at Strathclyde University and participated in a joint project with the Spinal Cord Injury Unit, Southern General Hospital, Glasgow, U.K. He joined the Hong Kong Polytechnic University in 1999 and as Professor in the Interdisciplinary Division of Biomedical Engineering in 2012. His research interests include rehabilitation robot, the control of functional electrical stimulation for upper and lower extremity functions, sensor development, stroke rat model and gait training rehabilitation on persons after stroke.
